# Be-dataHIVE: a base editing database

**DOI:** 10.1186/s12859-024-05898-0

**Published:** 2024-10-15

**Authors:** Lucas Schneider, Peter Minary

**Affiliations:** Department of Computer Science, University of Oxford, Parks Road, Oxford, OX1 3QD UK

## Abstract

Base editing is an enhanced gene editing approach that enables the precise transformation of single nucleotides and has the potential to cure rare diseases. The design process of base editors is labour-intensive and outcomes are not easily predictable. For any clinical use, base editing has to be accurate and efficient. Thus, any bystander mutations have to be minimized. In recent years, computational models to predict base editing outcomes have been developed. However, the overall robustness and performance of those models is limited. One way to improve the performance is to train models on a diverse, feature-rich, and large dataset, which does not exist for the base editing field. Hence, we develop BE-dataHIVE, a mySQL database that covers over 460,000 gRNA target combinations. The current version of BE-dataHIVE consists of data from five studies and is enriched with melting temperatures and energy terms. Furthermore, multiple different data structures for machine learning were computed and are directly available. The database can be accessed via our website https://be-datahive.com/ or API and is therefore suitable for practitioners and machine learning researchers.

## Introduction

Base editing is a genome engineering application that utilises the CRISPR-dCas toolbox [1]. The approach has the vast potential to transform healthcare and help cure rare diseases. The process to design highly efficient base editors for specific gene sections is resource intensive, and the editing outcome is often not easily predictable partly due to a wide range of factors base editing outcomes can depend on [2]. Another complicating factor is the occurrence of off-target effects [3], which have been also observed in traditional CRISPR-Cas9 based gene editing experiments. It was shown that the extent of the CRISPR-Cas9 based cleavage activity not only depends on the guide RNA and target DNA sequences but also on additional factors such as the GC content of the context sequence surrounding the target DNA and CRISPRspec-derived energy terms [4, 5]. These features among others were utilised when building deep learning models for off-target cleave activity prediction [5]. Therefore, it is expected that these types of features will also serve useful in building machine learning models for predicting base editing efficiency rates. In addition, bystander mutations can also lead to undesired outcomes that should be minimized. Therefore, base editing prediction models are needed to streamline the development of individualized base editors and to estimate any adverse mutations. Presently there are only a handful of models for predicting efficiency rates and bystander outcome rates (see for example [2, 3, 6–10]) that do no utilise all available data. For those interested in individual models, a more detailed discussion can be found in the Appendix. A unified and holistic dataset would be beneficial to build the next generation of base editing prediction models that are more robust to different experimental setups and perform better than the current ones. Therefore, we develop BE-dataHIVE, the first comprehensive database for base editing. The database is the biggest dataset to date with over 460,000 data points. Additionally, the database is enriched with melting temperatures, and energy terms that will be advantageous for building the next generation of deep learning models for predicting base editing efficiency rates and bystander outcome rates.

The remainder of this manuscript is structured as follows. An overview of the different base editing metrics is detailed in Sect. "Base editing prediction tasks". Section "Data acquisition and processing" describes the creation of the database and covers the data acquisition and processing. Our data enrichment approach is elaborated on in Sect. "Data enrichment". The computation of various data representations for machine learning is outlined in the Sect. "Data representation", which is followed by an overview of our technical implementation, including website, API interface, and Python wrapper. Following, a concrete machine learning use case is illustrated. Finally, we summarize our results. Additional insights about the database fields and data acquisition process are reported in the supplementary data.

## Materials and methods

### Base editing prediction tasks

In the base editing field, there exist two main computational tasks: the prediction of efficiency rates and bystander mutations (the latter entails the prediction of bystander edit rates or bystander outcome rates). In the following, we will define those tasks mathematically by using the denominations:

- *E*: Total number of reads
- $E_{{\text {edited}}}(s, e)$: Number of reads with at least one edit within the editing window starting at position *s* and ending at position *e* (e.g., bases 3 to 10)
- $E_{{\text {pos}}}(i)$: Number of edits at a specific position *i*
- $E_{{\text {outcome}}}(i, x, y)$: Number of edits at a specific position *i* that changed the underlying base *x* to base *y*
- ${\text {edit}}(i, k)$: Indicator function for an edit occurring at position *i* in read *k*, where ${\text {edit}}(i, k) = 1$ if an edit occurred, and ${\text {edit}}(i, k) = 0$ otherwise
- ${\text {outcome}}(i, k, x, y)$: Indicator function for an outcome change at position *i* in read *k* from base *x* to base *y*, where ${\text {outcome}}(i, k, x, y) = 1$ if such an outcome occurred, and ${\text {outcome}}(i, k, x, y) = 0$ otherwise
- *k*: Individual read *k*, where *k* ranges from 1 to *E*

*Efficiency rates* ($R_{{\text {eff}}}(s, e)$) are defined as the proportion of reads with edited outcomes within a certain editing window, such as between bases 3 (*s*) and 10 (*e*), of the target to total reads (see for example [7]). One can think of the editing window as a subsection of the target sequence where the editing activity is the strongest.

1 $$\begin{aligned} R_{{\text {eff}}}(s, e) = \frac{E_{{\text {edited}}}(s, e)}{E} \end{aligned}$$

To account for the fact that a read is counted as edited if any position within the window is edited, we can define $E_{{\text {edited}}}(s, e)$ as:

2 $$\begin{aligned} E_{{\text {edited}}}(s, e) = \sum _{k=1}^{E} \left[ 1 - \prod _{i=s}^{e} (1 - {\text {edit}}(i, k)) \right] \end{aligned}$$

where ${\text {edit}}(i, k) = 1$ if there is an edit at position $i$ in read $k$, and 0 otherwise.

The product term $\prod _{i=s}^{e} (1 - {\text {edit}}(i, k))$ evaluates to 0 if there is any position $i$ within the window $[s, e]$ where ${\text {edit}}(i, k) = 1$. If there are no edits at any position $i$ in read $k$, the product will be 1.

*Bystander edit rates* ($R_{{\text {bystander}}}(i)$) are defined as the number of edits at a given position (*i*) divided by total reads *E* while *bystander outcome rates* ($R_{{\text {outcome}}}(i, x, y)$) are defined as the number of edits at a specific position (*i*) that changed the underlying base *x* to base *y* divided by *E*. Thus, for the bystander tasks, there exist two possible forecasting targets – edit rates and outcome rates and both are typically expressed as editing fractions.

3 $$\begin{aligned} R_{{\text {bystander}}}(i) = \frac{E_{{\text {pos}}}(i)}{E} \end{aligned}$$

where $$E_{{\text {pos}}}(i) = \sum _{k=1}^{E} {\text {edit}}(i, k)$$.

4 $$\begin{aligned} R_{{\text {outcome}}}(i, x, y) = \frac{E_{{\text {outcome}}}(i, x, y)}{E} \end{aligned}$$

where $$E_{{\text {outcome}}}(i, x, y) = \sum _{k=1}^{E} {\text {outcome}}(i, k, x, y)$$.

Edit prediction solely provides information if a base change occurred at a certain position (e.g., an edit at position 3), while outcome forecasts are more granular and give insights into the resulting base change (e.g., an edit at position 3 where A $\rightarrow$ T), taking into account unexpected nucleotide alterations [3, 6–11].

Efficiency rates and bystander edit rates are closely related to each other. Efficiency rates are always smaller or equal to the sum of the bystander edit rates, which can be illustrated mathematically. Using Eq. 3, the sum of bystander edit rates over the editing positions from $s$ to $e$ is:

5 $$\begin{aligned} \sum _{i=s}^{e} R_{{\text {bystander}}}(i) = \sum _{i=s}^{e} \frac{E_{{\text {pos}}}(i)}{E} \end{aligned}$$

Substituting $$E_{{\text {pos}}}(i) = \sum _{k=1}^{E} {\text {edit}}(i, k)$$:

6 $$\begin{aligned} \sum _{i=s}^{e} R_{{\text {bystander}}}(i) = \sum _{i=s}^{e} \frac{\sum _{k=1}^{E} {\text {edit}}(i, k)}{E} \end{aligned}$$

Rearranging the summation:

7 $$\begin{aligned} \sum _{i=s}^{e} R_{{\text {bystander}}}(i) = \frac{1}{E} \sum _{k=1}^{E} \sum _{i=s}^{e} {\text {edit}}(i, k) \end{aligned}$$

By comparing Eqs. 1, 2, and 7, one can see that $\sum _{i=s}^{e} {\text {edit}}(i, k)$ will always be greater or equal to $\left[ 1 - \prod _{i=s}^{e} (1 - {\text {edit}}(i, k)) \right]$. Thus, the efficiency rate will also always be less than or equal to the sum of the bystander edit rates over the same positions:

8 $$\begin{aligned} R_{{\text {eff}}}(s, e) \le \sum _{i=s}^{e} R_{{\text {bystander}}}(i) \end{aligned}$$

As a more intuitive way to understand this inequality, think about $R_{{\text {eff}}}(s, e)$ as measuring the occurrence of at least one edit in the window, while $\sum _{i=s}^{e} R_{{\text {bystander}}}(i)$ is the sum of individual probabilities, which can sum to more than 1 if multiple edits are possible in the same read. Example calculations of efficiency and bystander rates can be seen in Sect. "Machine learning use case".

### Data acquisition and processing

To ensure the inclusion of as many studies as possible and an extensive database, we analysed 723 unique publications from the base editing field. All publications with “base editing”, “base editor” or “base editors” in the title were retrieved from Google Scholar. Following, the papers were analysed via Python for data sources that are reported in the individual studies and manually screened afterwards. Additional data points were requested for several studies to ensure a comprehensive dataset.

**Fig. 1:**
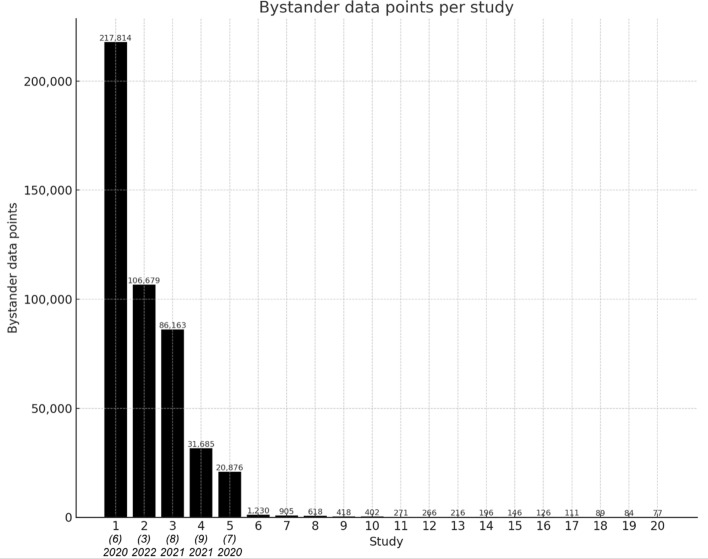
Available bystander data points per study in descending order

Figure 1 reports the available bystander data points per study in descending order. After five publications, there is a noticeable drop in available data points. In addition, subsequent studies often lack critical data points, such as total read counts or efficiency rates, and present data in formats that are challenging to standardize and extract, as these data points are usually embedded in the underlying data for tables and charts in publications. Considering the data quality and the fact that the first five studies account for over 98% of available data points, we establish a cut-off after the fifth article.

From a machine learning perspective, the small number of data points offered by the subsequent studies would not have a meaningful impact on model training. Machine learning models rely on large, high-quality datasets to generalize well. The robust dataset from the first five articles provides a strong foundation for model development. To further grow and diversify the database, we encourage researchers to submit their data via our homepage.

The included studies, along with selected key metrics, are reported in Table 1. Furthermore, stratification Table 2 shows selected metrics by base editors. Table 3 details key metrics and statistics segmented by studies.

For the data processing, we follow three steps. First, the raw data files for all studies are downloaded from the corresponding journal and unpacked using Python. Second, the data files are mapped to a common format where the bystander data is compressed to a single row, with editing outcomes at certain positions being represented by individual columns. Third, additional factors such as energy terms, and melting temperatures are incorporated in the database. All data processing was done in Python. The data processing script can be found under https://github.com/Lucas749/be-datahive. More details about the database format are reported in Table 1 in the supplementary data.

**Table 1:** Overview of studies included in the base editing database with selected metrics

References	Guides	Targets	Avg. efficiency rates full guide	Avg. bystander outcome rate	Avg. bystander edit rate	Cell lines	Base editors	Editor types
Arbab et al. (2020) [6]	33,280	33,612	0.291	0.703	0.054	HEK293T, U2OS, mES	ABE, ABE-CP1040, AID, BE4, BE4-CP1028, CDA, H47ES48A, T31A, T31AT44A, T44DS45A, eA3A, evoAPOBEC	ABE, CBE
Pallaseni et al. (2022) [3]	26,339	26,335	0.137	0.707	0.466	HEK293T, K562	ABE20m, ABE8e, ABERA, BE4-1, BE4-2, FNLS	ABE, CBE
Marquart et al. (2021) [9]	22,289	22,289	0.201	0.903	0.010	HEK293T	ABE8e, ABEmax, CBE4max, Target-AID	ABE, CBE
Yuan et al. (2021) [8]	13,660	13,660	–	0.356	0.014	HEK293T	A3G-CGBE, A3G-CTD-CGBE, BE3-WT, CBE4max, YE1-FNLS-BE3, YE1-FNLS-CGBE, eA3A-FNLS-CGBE, elegan-A3G-CTD-OPTI-CGBE, elegan-A3G-OPTI-CGBE, elegan-eA3A-OPTI-CGBE, elgan-OPTI-CGBE	CBE
Song et al. (2020) [7]	12,210	12,210	0.141	0.960	0.007	HEK293T	ABE, CBE	ABE, CBE

Please note that for some base editors certain metrics cannot be computed based on the underlying data and are therefore shown as –. For example, efficiency rates cannot be calculated based on bystander data alone as one could have multiple edits occurring at different positions within the same read (for a more detailed explanation please see Sect. "Base editing prediction tasks")

**Table 2:** Stratification of the database by base editors for selected metrics

Base editor	Unique guides	Unique targets	Unique PAMS	Avg. sequence length	Avg. length flanking sequence	Avg. efficiency rate full guide*	Avg. efficiency rate 3–10 window*
A3G-CGBE	7205	7205	38	79	59	–	–
A3G-CTD-CGBE	7022	7022	40	79	59	–	–
ABE	29,179	29,179	4	49.70	29.70	0.25	0.20
ABE20m	24,994	24,990	69	79	59	0.17	–
ABE8e	28,962	28,958	69	72.04	52.04	0.22	0.27
ABE-CP1040	16,295	16,295	4	56	36	0.26	–
ABEmax	8558	8558	–	20	–	0.26	0.25
ABERA	14,776	14,776	69	79	59	0.00	–
AID	18,876	18,876	4	56	36	0.41	–
BE3-WT	8522	8522	36	79	59	–	–
BE4	12,607	12,607	4	56	36	0.27	–
BE4-1	14,776	14,776	69	79	59	0.10	–
BE4-2	11,811	11,811	69	79	59	0.09	–
BE4-CP1028	13,534	13,534	4	56	36	0.21	–
CBE	10,221	10,221	4	30	10	0.08	0.07
CBE4max	16,881	16,881	31	46.46	26.46	0.18	0.14
CDA	17,306	17,306	4	56	36	0.31	–
eA3A	12,115	12,446	16	55.37	35.37	0.29	–
eA3A-FNLS-CGBE	7106	7106	33	79	59	–	–
elegan-A3G-CTD-OPTI-CGBE	6885	6885	39	79	59	–	–
elegan-A3G-OPTI-CGBE	7204	7204	36	79	59	–	–
elegan-eA3A-OPTI-CGBE	7369	7369	37	79	59	–	–
elgan-OPTI-CGBE	7921	7921	35	79	59	–	–
evoAPOBEC	16,733	16,733	4	56	36	0.29	–
FNLS	14,776	14,776	69	79	59	0.17	–
H47ES48A	7049	7049	4	56	36	0.33	–
T31A	827	879	16	54.06	34.06	0.22	–
T31AT44A	3089	3135	16	55.66	35.66	0.22	–
T44DS45A	1101	1101	4	56	36	0.19	–
Target-AID	10,177	10,177	–	20	–	0.13	0.05
YE1-FNLS-BE3	8021	8021	34	79	59	–	–
YE1-FNLS-CGBE	7040	7040	33	79	59	–	–

Please note that for some base editors certain metrics cannot be computed based on the underlying data and are therefore shown as –. For example, efficiency rates cannot be calculated based on bystander data alone as one could have multiple edits occurring at different positions within the same read (for a more detailed explanation please see Sect. "Base editing prediction tasks")

*Calculated rate is used if no efficiency rate is reported

**Table 3:** Stratification of the database by studies for selected metrics

Metric	Statistic	References				
		Arbab et al. (2020) [6]	Pallaseni et al. (2022) [3]	Marquart et al. (2021) [9]	Yuan et al. (2021) [8]	Song et al. (2020) [7]
Guide length	Value	20	20	20	20	20
Mismatch guide/sequence	Percentage	0.00	0.74	0.00	0.04	0.00
Sequence length	Min	40	40	20	20	30
	Average	40	40	20	20	30
	Max	34	40	20	20	30
Full context sequence	Min	35	79	20	79	30
	Average	56	79	20	79	30
	Max	61	79	20	79	30
Flanking sequence length	Min	41	59	0	59	10
	Average	36	59	0	59	10
	Max	15	59	0	59	10
Total reads experiment	Min	101	3	100	0	110
	Average	4527	6353	1999	0	2422
	Max	688,505	337,154	285,776	0	1,156,803
Edited count experiment	Min	100	0	0	0	0
	Average	1028	0	1419	0	517
	Max	106,103	0	49,761	0	314,275
Efficiency rate full guide*	Min	0.00	0.00	0.03	0.00	0.00
	Average	0.29	0.14	0.20	0.00	0.00
	Max	1.00	1.00	1.00	0.00	0.00
Efficiency Rate 3–10 Window*	Min	0.00	0.00	0.00	0.00	0.00
	Average	0.00	0.00	0.15	0.00	0.00
	Max	0.00	0.00	1.00	0.00	0.00

*Calculated rate is used if no efficiency rate is reported

### Data enrichment

#### Physical energy terms

In line with Störtz and Minary [12], we add various interaction energies figures to the database to enrich the dataset. Based on Alkan et al.’s [13] approximate energy model for Cas9-gRNA-DNA binding, we compute multiple energy terms. In total, 24 energy figures are added to the database based on different parameter combinations (see supplementary Table 2 for an overview). Furthermore, we use RNAfold [14] to compute the minimum free energy (MFE) secondary structure of the gRNA sequence.

Although these energy metrics were originally developed for traditional Cas9 systems, base editing leverages a modified, inactive Cas protein, known as dead Cas (dCas), which does not cleave DNA. Despite this modification, we hypothesize that these energy terms will still enhance the predictive power of machine learning models in the field of base editing. The incorporation of these energy terms provides several advantages:

- *Enhanced Predictive Accuracy*: Energy terms offer quantitative insights into the stability and efficiency of gRNA-DNA binding interactions, potentially allowing models to better forecast the likelihood of successful base editing events.
- *Robustness Across gRNA Variations*: Energy terms help models generalize across different gRNA sequences and target sites by providing a consistent measure of interaction strength and stability, thereby potentially enhancing model robustness.

Future research will further investigate the importance and impact of these energy features on the performance of machine learning models for base editing.

#### Melting temperature

In addition, we add the melting temperatures of the 20nt target sequence and gRNA to the database. The melting temperature is computed via the Biopython MeltingTemp module [15] using the $Tm\_NN$ function which calculates the temperature based on nearest neighbour thermodynamics and corrects amongst others for mismatches, dangling ends, and salt concentration. Melting temperatures find usage in some base editing prediction models, such as Pallaseni et al. [3] and Arbab et al. [6].

### Data representation

There exist many methods to encode sequence data and process information efficiently. Besides traditional methods, such as one-hot encoding or k-mers, novel approaches such as BASiNET [16] and Hilbert curve encodings [17, 18] have been developed. Our database is designed to easily integrate any type of encoding method, independently of the underlying complexity, and can be extended with more encoding techniques.

Currently, the database supports two main encoding methods: one-hot encoding and Hilbert curve encoding. We chose these two approaches to illustrate the flexibility and capability of our database and to offer users one standard encoding approach (one-hot encoding) and a more novel and complex encoding framework from the field of imaging (Hilbert curve encoding) that has produced promising results in machine learning models [17, 18].

#### One-hot encoding

One-hot encoding is a commonly used preprocessing method to convert categorical data into a format that can be understood by machine learning algorithms. Each category value is converted into a new binary feature that takes a value of 1 for its respective category and 0 for others. In a DNA sequence context, nucleotides ’A’, ’T’, ’C’, ’G’ can be one-hot encoded as [1,0,0,0], [0,1,0,0], [0,0,1,0], [0,0,0,1] respectively. This method efficiently represents categorical data.

#### Hilbert curve encoding

Sequences are represented as a Hilbert curve image [19]. This imaging approach is used in converting multi-dimensional data into one-dimensional data while preserving locality, meaning that points that are close in higher dimensions remain close when mapped to the Hilbert curve. Using Hilbert curves allows DNA sequences to be represented in a two-dimensional space while preserving the locality of the nucleotides. Each point on the Hilbert curve corresponds to a specific nucleotide in the sequence. The curve covers every point in a square grid with a size of any power of 2. A practical example of the generation of a Hilbert curve encoding can be found in the Appendix. For a detailed explanation on Hilbert curves and the exact construction methodology we refer to Anjum et al. [17].

### Technical implementation

**Fig. 2:**
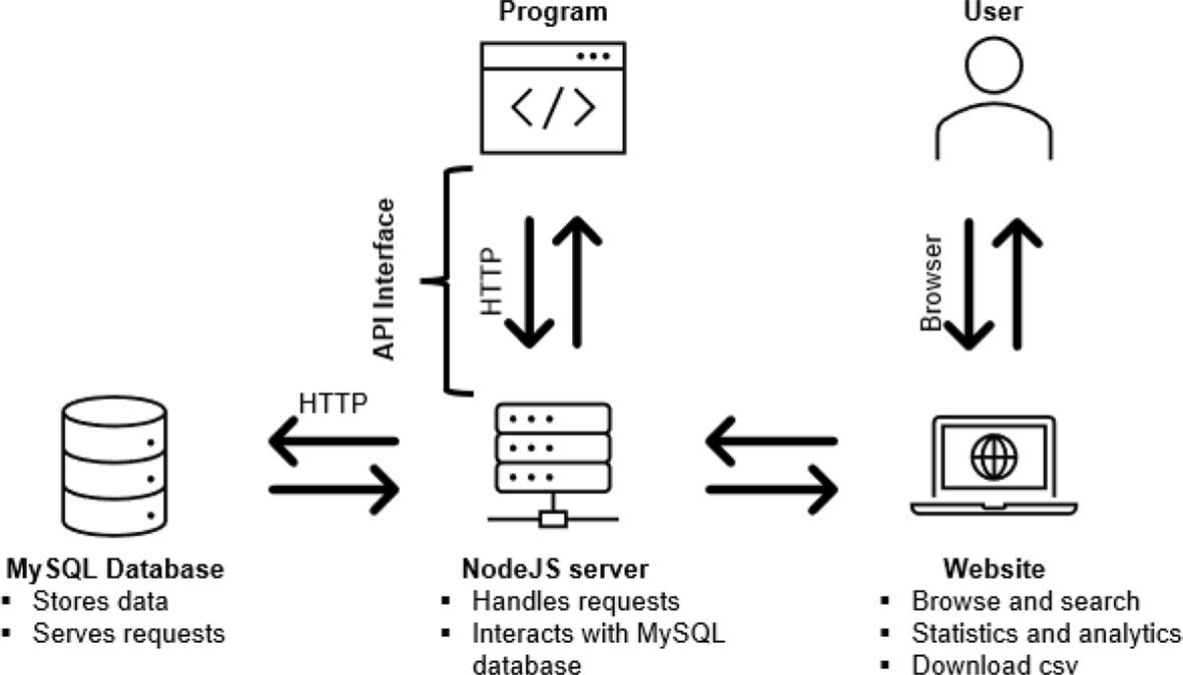
Illustration of the technical implementation of BE-dataHIVE

The database consists of four main components, a mySQL database, a Node.js server for REST API queries, a Python wrapper for the API, and our website https://be-datahive.com/. The setup is illustrated in Fig. 2. The REST server, utilizing Node.js as runtime framework, provides data for the website and can also be directly accessed from users to serve individual queries. The website is written from scratch using CSS, HTML, and JavaScript. API calls to the database are done via JavaScript’s asynchronous fetch method.

Our setup enables highly individualized and fast data queries and offers users two interfaces - our website and API. Furthermore, the framework is easily expandable to accommodate and incorporate various data views, especially for machine learning applications.

#### Website interface

**Fig. 3:**
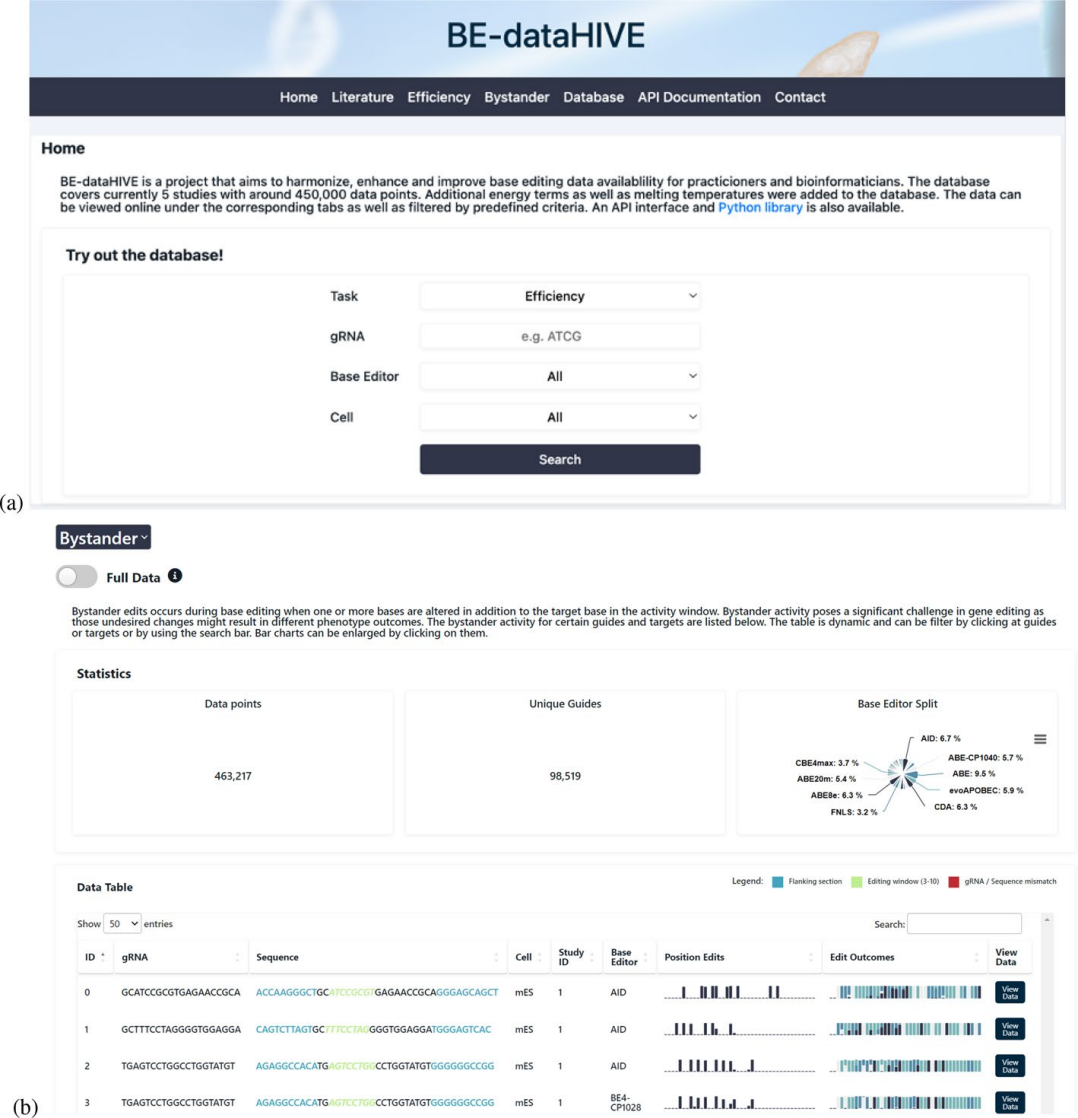
Web interface of BE-dataHIVE. Experiments can be filtered on the home screen (**a**) and bystander data can be examined (**b**)

Our website https://be-datahive.com/ provides a convenient way for practitioners to look up guides, targets, and base editor efficiency rates as well as bystander outcomes (see Fig. 3). For example, if a lab would like to investigate the bystander activity of a certain guide RNA, they can search for the specific guide but also for similar sequences via the search feature on our web page.

The website offers the following features:

- Browsing and searching by gRNA, base editor, and cell lines
- Access to statistics and analytics for bystander and efficiency data
- Charting of data
- Direct csv download
- API to interact with the mySQL database for customised data requests

#### Application programming interface

The database can be accessed via a REST-API that enables easy access to the underlying data as well as a flexible way to interact with the database. The data can be directly filtered and modified via the API and accessed from any programming language that supports http requests. The API will be particularly relevant for data scientists and machine learning researchers as it provides the flexibility to filter and retrieve the desired data directly from the server without any intermediary steps. The API documentation can be found under https://be-datahive.com/documentation.html.

#### Python API wrapper

To provide a simple way to obtain data directly in Python, a widely used programming language for machine learning, we wrote the Python library be_datahive that handles all API requests and data handling. Data can be retrieved directly via the package. Furthermore, the wrapper implements some basic machine learning data handling routines, such as the creation of a labelled dataset. be_datahive is available on GitHub and PyPi. Detailed usage examples are showcased on our GitHub.

### Machine learning use case

Base editing features two types of prediction tasks, namely efficiency rate and bystander predictions. The former predicts the overall editing efficiency while the latter aims at predicting bystander edit rates or the more informative bystander outcome rates (as defined above). Easy access to training data for both types of prediction tasks can be facilitated by BE-dataHIVE. The required machine learning ready data can simply be obtained and multiple feature encodings are available out-of-the-box. Python users can use our Python API wrapper that returns the requested data in a few lines of code. Practical coding examples for training machine learning models with our database are available on our Python wrapper GitHub. The following section illustrates the structure of one data point based on a specific example to make it easier for readers to grasp the data format. Assuming that one labelled data point has the format (*X*, *y*), where *y* is the prediction target one aims to predict based on *X*, which typically denotes the features. Data points for efficiency rate, bystander edit rate and bystander outcome rate differ in terms of *y* so here we give an example data point for each rate that shares the same *X* illustrated in Eq. 9. Using our database, we retrieve 33 features for *X*, such as melting temperatures, energy terms, and gRNA. One-hot and Hilbert curve encodings are available for all nucleic acid sequence fields.

9 $$ X = \left[ {\begin{array}{*{20}l}    {{\text{gRNA}}} \hfill  \\    {{\text{Pam Sequence}}} \hfill  \\    {{\text{Full Context Sequence Padded}}} \hfill  \\    {{\text{gRNA Sequence Match}}} \hfill  \\    {{\text{Cell}}} \hfill  \\    {{\text{Base Editor}}} \hfill  \\    {{\text{Melt Temperature gRNA}}} \hfill  \\    {{\text{Melt Temperature Target}}} \hfill  \\    {{\text{Energy 1}}} \hfill  \\     \vdots  \hfill  \\    {{\text{Energy 24}}} \hfill  \\    {{\text{Free Energy}}} \hfill  \\   \end{array} } \right] = \left[ {\begin{array}{*{20}l}    {{\text{GGACCGTCGAAAATGGGCCT}}} \hfill  \\    {{\text{GGG}}} \hfill  \\    {{\text{NTCCAATATC}}...} \hfill  \\    {{\text{TRUE}}} \hfill  \\    {{\text{K562}}} \hfill  \\    {{\text{BE4 - 1}}} \hfill  \\    {56.96} \hfill  \\    {56.96} \hfill  \\    0 \hfill  \\     \vdots  \hfill  \\    {27} \hfill  \\    { - 4.6} \hfill  \\   \end{array} } \right]{\text{ }} $$

#### Efficiency rate prediction task

The efficiency data can be obtained by calling the endpoint “efficiency” in our API or Python wrapper. In the efficiency rate prediction task, we are forecasting a single number, which for our example (*X*) is

10 $$\begin{aligned} y = \begin{bmatrix} \text {Efficiency Full gRNA Reported} \\ \end{bmatrix} = \begin{bmatrix} 0.9840 \\ \end{bmatrix} \end{aligned}$$

#### Bystander edit rate prediction task

Endpoint “bystander” yields bystander edit and outcome data (see below). For bystander edit rate prediction the target matrix is of size $1 \times m$, containing editing fractions (number of edits divided by total reads) for every position. In our data point $m = 42$ and positions are determined relative to the start of the gRNA, meaning that position $-1$ would indicate the base immediately before the start of the gRNA sequence (see Eq. 11). In the case that only a single edit per base occurs per sample, we could simply sum up the vector to calculate the efficiency rate. However, multiple edits can occur at different positions within the same read, weaking this link between efficiency and bystander edit rate (see Eq. 8).11

#### Bystander outcome rate prediction task

Bystander outcome forecasts have as target a matrix of size $n \times m$, containing editing fractions (number of edits divided by total reads) for position and outcome combinations. *m* refers to the editing positions around the target sequence while *n* represents the possible editing outcomes A, T, C, or G for outcome predictions. Therefore, the matrix has four rows ($n=4$). Equation 12 shows the example, *y* for editing outcomes, which is a 4 × 42 matrix. Based on the example data, we can see that in 0.18% of the total reads the base at position − 9 is edited to an A nucleotide.12To demonstrate the utility of our database and the significance of newly incorporated features, such as energy terms and melting temperatures, we train a machine learning model to predict efficiency rates using various feature combinations. We employ a Gradient Boosting Regression model with a learning rate of 0.1, maximum depth of 3, and 100 boosting stages. The model is trained on $80\%$ of the dataset based on a five-fold cross-validation approach with Spearman correlation as the loss function. We use the same model and training approach while varying feature combinations. We create the following feature groups and test all four combinations:

1. *Baseline* Includes only one-hot encoded gRNA and full context sequence.
2. *Energy Terms* Includes all physical energy terms for the sequence and gRNA.
3. *Melting Temperature* Includes all melting temperatures for the sequence and gRNA.

**Fig. 4:**
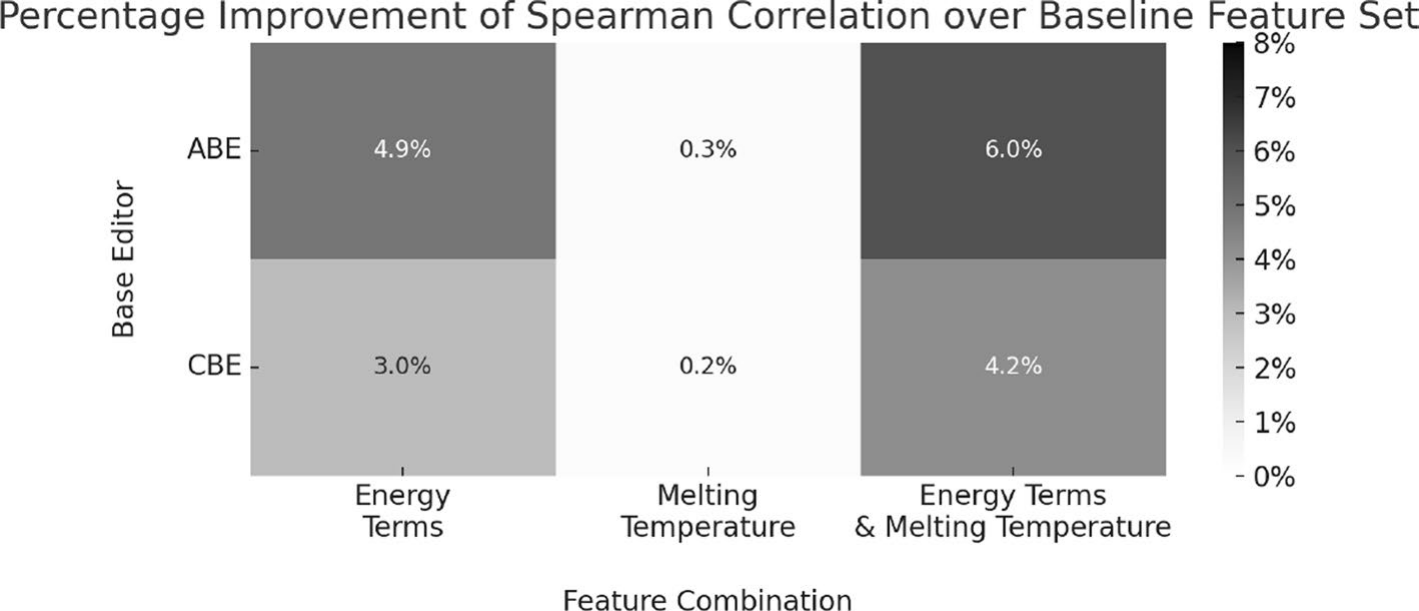
Percentage improvement of the Spearman correlation over the baseline feature set with respect to various feature combinations

Figure 4 shows the improvement of different feature combinations over the baseline model using Spearman correlation to assess performance. Models with an enhanced feature set outperform the baseline across ABEs and CBEs. The greatest performance improvements—$6.0\%$ higher Spearman correlation for ABEs and a $4.2\%$ increase for CBEs compared to the baseline—are achieved using both energy terms and melting temperatures. Using energy terms alone improves performance for ABEs and CBEs by $4.9\%$ and $3.0\%$, respectively. Melting temperatures increase the correlation by $0.3\%$ for ABEs and $0.2\%$ for CBEs. Overall, incorporating energy terms and melting temperatures into the feature set results in a substantial increase in Spearman correlation across ABE and CBE base editors. This systematic case study highlights the potential of the newly added features in our database to enhance the predictive power of base editing models.

## Results and discussion

We devise a base editing database that consists of over 460,000 individual data points, covering 32 distinct base editors and over 98,000 unique guides. BE-dataHIVE addresses multiple issues in the computational pipeline for base editing prediction models.

We create the first baseline dataset for base editing prediction tasks. The data is standardized and enriched with additional metrics from various data sources. Our dataset can be easily used by non-domain experts, significantly lowering the entry barrier for computer scientists and machine learning researchers to work on base editing outcome predictions. Similar to how standardized datasets like the Protein-Protein Interactions (PPI) [20] or MNIST [21] datasets facilitated advancements in their respective fields, we hope that our data will serve this crucial role for base editing.

In addition to compiling the first comprehensive collection of data points for base editing, we further enhance the underlying data with energy terms and melting temperatures that will allow more robust machine learning models.

Furthermore, our interactive web interface provides analytics for single data points that give researchers the possibility to look up relevant gRNA and target combinations as well as understand the underlying data points.

Moreover, our dataset is easily accessible via our web interface and API that allows flexible data queries for researchers to directly integrate base editing data into their studies. With our Python wrapper, users can create machine learning models with a few lines of code and access machine readable encodings (please see our Python wrapper GitHub for examples). Those generated encodings alone have a size of 72GB.

We hope the database will contribute to the literature to produce stronger base editing prediction models that are more robust and help streamline the efficient development of base editing systems to ultimately cure rare diseases caused by point mutations. We highly encourage laboratories and practitioners to reach out and submit base editing data to grow the database further and advance the field together.

## Supplementary Information

Supplementary Material 1.

## Figures and Tables

**Fig. 5:**
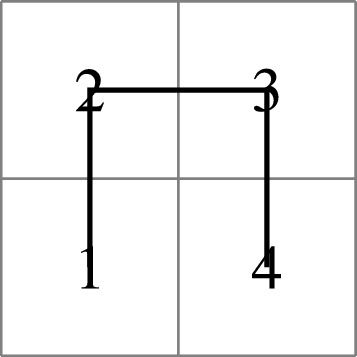
Illustration of a 2 × 2 Hilbert curve

**Fig. 6:**
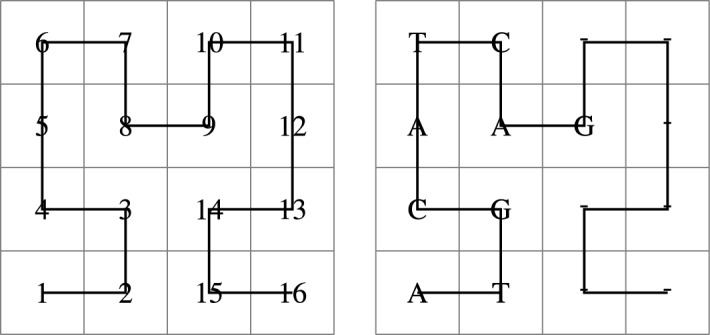
Mapping of sequence “ATGCATCAG” to a 4 × 4 Hilbert curve. Left shows the ordering of the Hilbert curve across the 4 × 4, while the right depicts the example sequence inside the grid

## Data Availability

The BE-dataHive database is available at https://be-datahive.com/. Users are not required to log in to access any of the database features.

## Appendix

### Base editing prediction models

In this section, we delve deeper into the current systems used to forecast base editing outcomes. There are seven core studies (see Table 4). While most publications utilise a machine learning framework, Dandage et al. [10] is the oldest and only article that relies on a deterministic workflow to estimate efficiency rates. From the machine learning models, neural networks are the most popular architecture types – four of the six publications build neural networks while the other two use tree based models. Most of the machine learning models focus on simple architectures, such as gradient boosted trees or logistic regressions [3, 6, 11]. Marquart et al. [9] is the only publication that enriches their own dataset with some of the data from Arbab et al. [6] and Song et al. [7]. Noticeable is that some models utilise different architectures within itself for different base editing prediction tasks. For example, the BE-Hive model uses gradient boosted trees for efficiency forecasts but a two-layer neural network for bystander predictions. Amongst the publications, there seems to be a slight bias towards predicting CBE outcomes as 100% of articles cover those, while solely 71% support ABEs. However, most studies incorporate ABEs and CBEs. Overall, academia developed six models for base editing outcome predictions, namely beditor, BE-Hive, DeepBaseEditor, BE-SMART, BE-DICT, and FORECasT-BE.

**Table 4:** Overview of the state-of-the-art prediction models in the literature

References	Year	Task	Model approach	Architecture	Model	Model details	ABE support	CBE support
Dandage et al. [10]	2019	Efficiency	Deterministic	N/A	beditor	Computational scoring system that uses the Burrows-Wheeler aligner to determine mismatches and apply different penalty scores if a mismatch is near the PAM, genic or intergenic	✓	✓
Arbab et al. [6]	2020	Efficiency	Machine learning	Decision tree	BE-Hive	Gradient boosted regression trees	✓	✓
		Bystander		Neural network		Deep conditional autoregressive machine learning model with encoder/decoder. Encoder has two hidden layers and the decoder exhibits five hidden layers. The network is fully connected		
Song et al. [7]	2020	Efficiency and bystander	Machine learning	Neural network	DeepBaseEditor (DeepABE/DeepCBE)	Two to three hidden layer deep neural network with convolution and dropouts	✓	✓
Koblan et al. [11]	2021	Efficiency and bystander	Machine learning	Neural network, regression, decision tree	Mixed	BE-Hive, logistic regression, gradient boosted regression trees	✗	✓
Yuan et al. [8]	2021	Bystander	Machine learning	Neural network	BE-SMART	Deep neural network model with dropout	✗	✓
Marquart et al. [9]	2021	Efficiency	Machine learning	Neural network	BE-DICT	Attention-based deep neural network with an encoder block that has a self-attention layer, layer normalization and residual connections, and a feed forward network	✓	✓
		Bystander				Extension of the efficiency model. Encoded block of the efficiency module feeds into an encoder-decoder attention layer together with positional embeddings		
Pallaseni et al. [3]	2022	Efficiency and bystander	Machine learning	Decision tree	FORECasT-BE	Gradient boosted regression trees	✓	✓

### Hilbert curve encoding example

In this section, we will construct a practical example of the Hilbert curve encoding based on a sequence of nucleotides. Assume we want to encode the sequence “ATGCATCAG”. In order to fit the full sequence of length 9 into a matrix, we need at least the dimension 4 × 4. First, we start with the simplest 2 × 2 Hilbert curve that has the following shape (see Fig. 5).

Based on the 2 × 2 Hilbert curve we can expand the grid to a 4 × 4 matrix (see Fig. 6). The nucleotides can now be placed into the grid in the Hilbert curve order.

The individual letter can be further encoded, with for example one-hot encoding. This transforms the 4 × 4 matrix into a three dimensional matrix of the shape 4 × 4 × 4 that is preserving the locality of the data. The Hilbert curves are constructed in line with Anjum et al. [17], which build a CNN model for enhancer predictions utilising this graphical representation. For a detailed explanation on Hilbert curves and the exact construction methodology we refer to Anjum et al. [17].
